# Research on the targeted improvement of the yield of a new VB_12_-producing strain, *Ensifer adhaerens* S305, based on genomic and transcriptomic analysis

**DOI:** 10.1186/s12896-023-00824-3

**Published:** 2023-12-11

**Authors:** Yongheng Liu, Wei Huang, Qi Wang, Cilang Ma, Yongyong Chang, Jianyu Su

**Affiliations:** School of Life Science, Ningxia University, Xixia District, No. 539, Helan Moutain-West Road, Yinchuan, 750021 Ningxia China

## Abstract

**Background:**

Vitamin B_12_ (VB_12_) has a wide range of applications and high economic value. In this study, a new strain with high VB_12_ production potential, *Ensifer adhaerens* S305, was identified in sewage. Because *E. adhaerens* strains have become the main strains for VB_12_ production via fermentation in recent years, the directional modification of the S305 strain to obtain a strain suitable for the industrial production of VB_12_ has great potential and commercial value.

**Results:**

*16S rRNA* and genome-wide phylogenetic tree analysis combined with average nucleotide identity (ANI) analysis showed that the high-yielding VB_12_ strain was a *E. adhaerens* strain and that its VB_12_ synthesis pathway genes were highly similar to related genes of strains of this and other species, including *E. adhaerens* Casida A, *Pseudomonas denitrificans* SC 510, and *E. adhaerens* Corn53. High-pressure liquid chromatography (HPLC) results indicated that the VB_12_ yields of the S305 strain were more than double those of the Casida A strain under different medium components. Multiple genes with significantly upregulated and downregulated transcription were identified by comparing the transcription intensity of different genes through transcriptome sequencing. KEGG enrichment analysis of the porphyrin metabolism pathway identified 9 significantly upregulated and downregulated differentially expressed genes (DEGs) in the VB_12_ synthesis pathway, including 7 transcriptionally upregulated genes (*cobA*, *cobT*, *hemA*, *cobJ*, *cobN*, *cobR*, and *cobP*) that were episomally overexpressed in the Casida A strain. The results showed that the VB_12_ yield of the overexpressed strain was higher than that of the wild-type strain. Notably, the strains overexpressing the *cobA* and *cobT* genes exhibited the most significant increases in VB_12_ yield, i.e., 31.4% and 24.8%, respectively. The VB_12_ yield of the S305 strain in shake-flask culture was improved from 176.6 ± 8.21 mg/L to 245.6 ± 4.36 mg/L by integrating the *cobA* and *cobT* genes into the strain.

**Conclusion:**

Phylogenetic tree and ANI analysis showed that the *Ensifer* and *Sinorhizobium* strains were quite different at the genome level; the overexpression and integrated expression of significantly upregulated genes in the VB_12_ synthesis pathway could increase the yield of VB_12_, further improving the VB_12_ yield of the *E. adhaerens* S305 strain.

**Supplementary Information:**

The online version contains supplementary material available at 10.1186/s12896-023-00824-3.

## Introduction

Vitamin B_12_ (VB_12_), also known as cobalamin, is the only metal-containing vitamin that is essential for vertebrates. The main physiological effect of VB_12_ is the inhibition of the occurrence of pernicious anaemia in animals. This vitamin is mainly used in medical treatments and as a feed or food additive. Due to its complex chemical structure and extremely cumbersome production using chemical synthesis methods, VB_12_ is produced by microbial fermentation. Reportedly, the microorganisms that produce VB_12_ mainly include *Propionibacterium freudenreichii* (*P. freudenreichii*) [1], *Pseudomonas denitrificans* (*P. denitrificans*) [2], *Ensifer adhaerens* (*E. adhaerens*) [3], and *Sinorhizobium meliloti* (*S. meliloti*) [4]. Among them, *Propionibacterium* strains have been certified as food-safe strains by the U.S. Food and Drug Administration (FDA) [5], and *P. denitrificans* was the first high-yielding strain used for genetic modification [6, 7]. However, the production of VB_12_ by *Propionibacterium* is complex, yielding many byproducts, and the production of VB_12_ by *P. denitrificans* requires a long cycle and low bacterial density [8]. Compared with the above two types of bacteria, *E. adhaerens* has a shorter production cycle and fewer byproducts; therefore, it has become the main strain for VB_12_ production in recent years. Research on the production of VB_12_ by *E. adhaerens* has only been reported in recent years. HOAN THI VU et al. identified the VB_12_-producing strain *E. adhaerens* CS8α. Gene sequencing results showed that the strain has 22 *cob* genes. *Rhizobium* strains (such as *Rhizobium leguminosarum* WSM2304) also have these *cob* genes; therefore, it is speculated that *Rhizobium* and *E. adhaerens* have the ability to biosynthesize VB_12_ [9]. The European Food Safety Authority (EFSA) evaluated the safety of *E. adhaerens* CICC 11008 s with regard to VB_12_ production, and the results indicated that *E. adhaerens* strains are safe and reliable VB_12_ producers [10, 11].

**Fig. 1:**
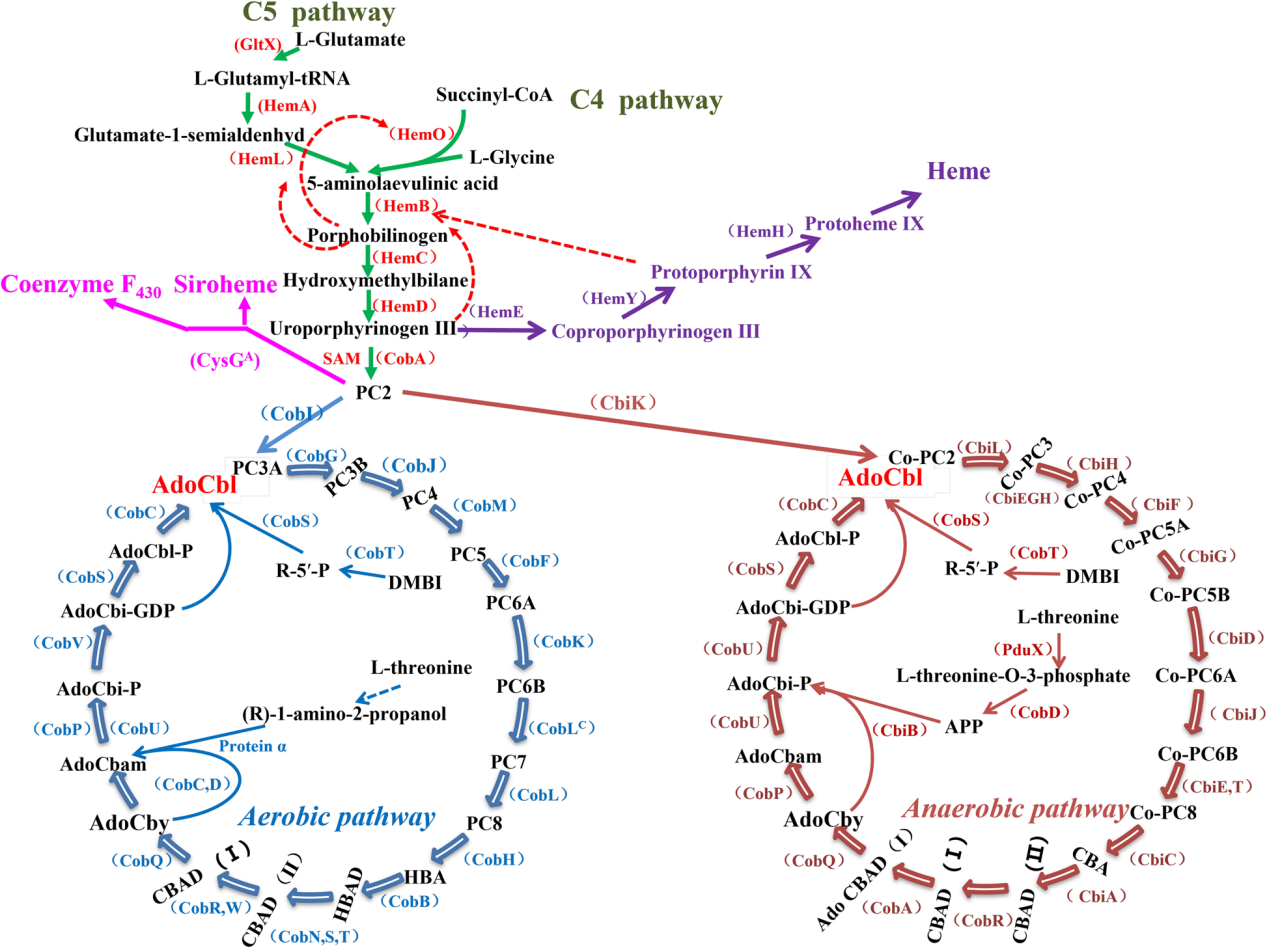
Biosynthetic metabolic pathway for VB_12._ The green arrow in the figure represents a common pathway of VB_12_ synthesis, the red dashed arrow represents feedback inhibition, the purple arrow and magenta arrow represent the two main metabolic pathway branches for VB_12_, the blue circle represents the aerobic pathway, and the red circle represents the anaerobic pathway. The abbreviations and full names of some metabolites in the figure are shown at the end of the article. The abbreviations in parentheses represent the enzymes in each step

As shown in Fig. 1, the biosynthesis of VB_12_ is very complex, exhibiting a bicyclic pathway; there are approximately 30 enzymes involved in its de novo synthesis. Early scholars divided the biosynthesis pathway of VB_12_ into an anaerobic synthesis pathway (the blue circle on the left side of Fig. 1) and an aerobic synthesis pathway (the red circle on the right side of Fig. 1) on the basis of the dependence of microorganisms on oxygen. Later, through an in-depth comparative study of these two synthesis pathways, the main difference between the two was identified as the chelation time of cobalt ions and the enzymes that catalyse most reactions [12]. The pathway for synthesizing 5-aminolevulinic acid from glutamic acid, i.e., the C5 pathway, is a common pathway that exists in most microorganisms, such as *E. adhaerens*, the object of this study. The pathway for synthesizing 5-aminolevulinic acid from glycine, i.e., the C4 pathway, mainly exists in some bacteria in the class Alphaproteobacteria in the phylum Proteobacteria [13, 14]. A new VB_12_-producing strain was discovered in this study. The strain was identified by genome bioinformatics analysis and named *E. adhaerens* S305 (hereinafter referred to as the S305 strain). High-pressure liquid chromatography (HPLC) was used to preliminarily assess the VB_12_ production capacity of the S305 strain and an *E. adhaerens* strain, *E. adhaerens* Casida A (ATCC 33212, hereinafter referred to as the Casida A strain). Considering the significant difference in yield between the two strains, the transcription of the two strains cultured under the same conditions for different durations was compared and analysed through genome and transcriptome sequencing. Episomal overexpression of the key genes was preliminarily verified, and finally, the yield of VB_12_ from the S305 strain was greatly improved through gene integration expression. Therefore, this study provides a new reference for the classification of *Ensifer* and *Sinorhizobium* species and the genetic modification of VB_12_-producing strains.

## Results

### Basic morphology, genome characteristics, and identification of the S305 strain

**Fig. 2:**
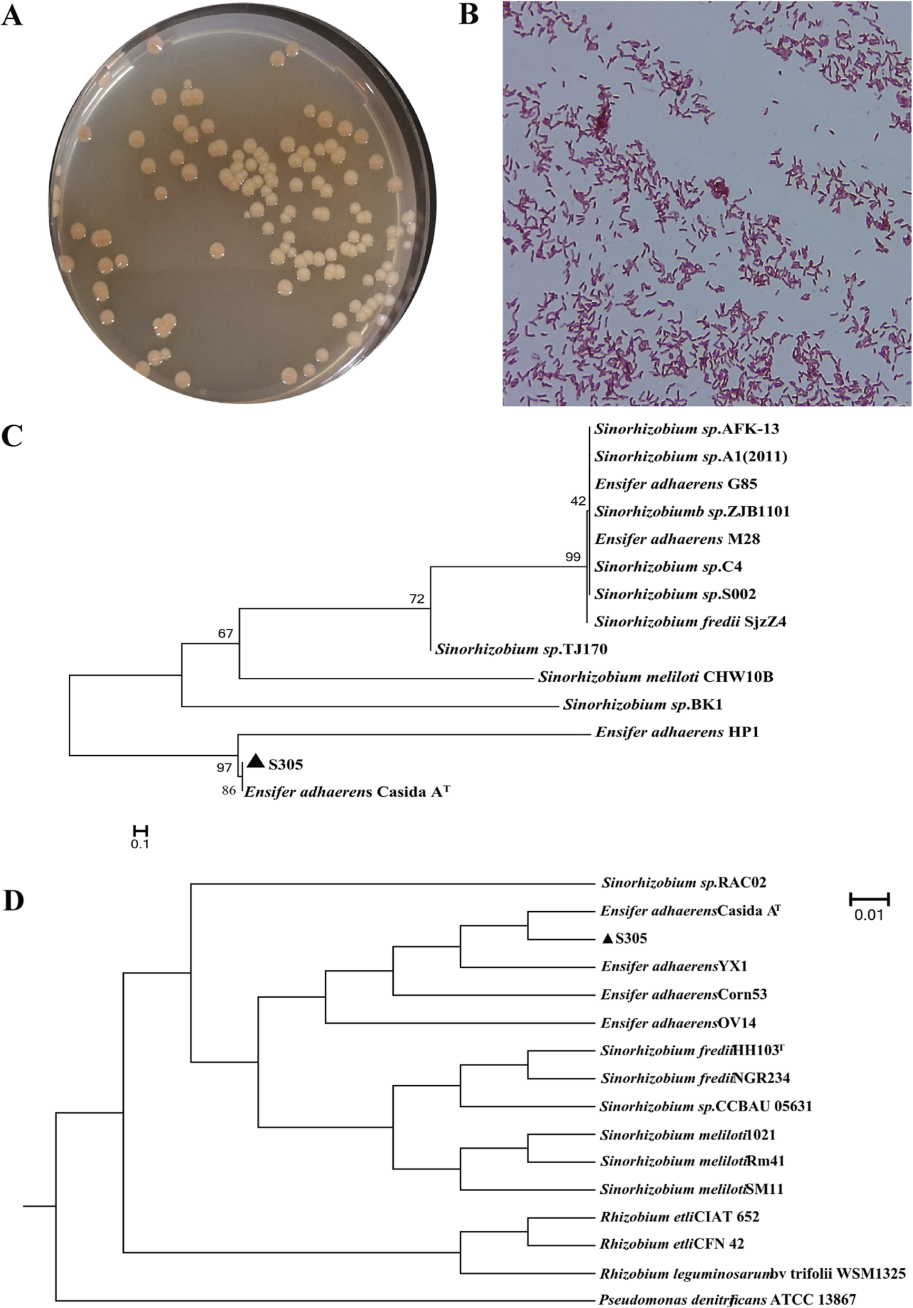
Basic growth morphology and molecular identification of the S305 strain. **A** Colony morphology of S305 on solid plates. **B** Microscopy examination of the S305 strain with Gram staining. The image was obtained at 1000 × magnification (objective oil lens: 100x; eye piece: 10 × magnification). **C** Phylogenetic evolutionary tree of the S305 16S rRNA constructed using the neighbour joining (NJ) method. The sequences aligned were *16S rRNA* (DQ911548.1) from *Sinorhizobium sp.* AFK-13; 16S rDNA (HQ288937.1) from *Sinorhizobium sp.* A1 (2011); *16S rRNA* (KR819181.1) from *Ensifer adhaerens* G85; *16S rRNA* (JQ927221.1) from *Sinorhizobium sp.* ZJB1101; *16S rRNA* (KC934865.1) from *Ensifer adhaerens* M28; *16S rRNA* (AF227753.1) from *Sinorhizobium sp.* C4; *16S rRNA* (AF285962.1) from *Sinorhizobium sp.* S002; *16S rRNA* (DQ786804.1) from *Sinorhizobium fredii* SjzZ4; *16S rRNA* (AJ505297.1) from *Sinorhizobium sp.* TJ170; *16S rRNA* (JQ322555.1) from *Sinorhizobium meliloti* CHW10B; *16S rRNA* (AJ012210.1) from *Sinorhizobium sp.* BK1; *16S rRNA* (MN083303.1) from *Ensifer adhaerens* HP1; *16S rRNA* (MW800616.1) from *Ensifer adhaerens* S305; and *16S rRNA* (CP015880.1) from *Ensifer adhaerens* Casida A. The numbers on the branches represents the self-expansion value, the branch clustering and length represent the phylogenetic relationships, and a "T" on the top right of the bacterial species name indicates that the strain is a representative species of the genus. **D** Phylogenetic evolutionary tree based on the entire genome of S305 and other strains. The genome sequences aligned were as follows: NZ_CP016450.1 from *Sinorhizobium sp.* RAC02; GCA_000697965.2 from *Ensifer adhaerens* Casida A; GCA_029439625.1 from *Ensifer adhaerens* S305; NZ_SSBU01000001.11 from *Ensifer adhaerens* YX1;GCA_009883655.1 from *Ensifer adhaerens* Corn53; NZ_CP007236.1 from *Ensifer adhaerens* OV14; NC_016812.1 from *Sinorhizobium fredii* HH103; NC_012587.1 from *Sinorhizobium fredii* NGR234; NZ_CP023063.1 from *Sinorhizobium sp.* CCBAU 05631; GCA_000006965.1 from *Sinorhizobium meliloti* 1021; GCA_002197045.1 from *Sinorhizobium meliloti* RM41; GCA_000218265.1 from *Sinorhizobium meliloti* SM11; GOLD: Go0003237 from *Rhizobium etli* CIAT 652; GOLD: Go0000395 from *Rhizobium etli* CFN 42; GOLD: Go0001809 from *Rhizobium leguminosarum* bv trifolii WSM1325; NZ_CP004143.1 from *Pseudomonas denitrificans* ATCC 13867. Branch clustering and length represent phylogenetic relationships, and a "T" on the top right of the strain name indicates that the strain is a representative strain of the genus

A total of 23 different bacterial strains were screened from sewage. Compared with other strains, the S305 strain was slightly red with a round, smooth, and sticky gram-negative colony (Fig. 2A) and a microscopically short rod-like shape (Fig. 2B) after being cultured in No. 1 solid medium (Supplementary methods 1) for 72 h. Analysis of the whole-genome sequencing results showed that the genome of the S305 strain (GenBank assembly accession GCA_029439625.1) has a total length of 7,365,904 bp, a GC content of 62.34%, and two superlarge plasmids. The predicted number of coding sequences (CDSs) in the chromosomal genome is 6906, and the average gene length is 928 bp, containing 62 tRNA genes, 15 rRNA genes, 53 ncRNA genes and 3 pseudogenes. NCBI genome alignment showed that the sequence of the S305 strain is similar to those of the Casida A strain (GenBank assembly accession GCA_029674665.1) and the *E. adhaerens* Corn53 strain (GenBank assembly accession GCA_009883655.1). A comparison of basic genome information showed that the three strains each had a chromosome and 2 plasmids, with similar GC contents in all three (Table S1). The results of a genome collinearity analysis showed that the genome sequences of the S305 strain had better collinearity with those of the Casida A strain than with those of other strains and that the S305 strain had a higher level of homology with the *E. adhaerens* strain (Fig. S1). A phylogenetic tree was constructed using the 16S rRNA sequence and whole-genome sequence of the S305 strain (Fig. 2C, D). The results further indicated that S305 was a *Ensifer* species; in addition, the average nucleotide identity (ANI) analysis showed that the highest ANI value of the S305 and Casida A strains was 99.09% (Fig. S2). An ANI value greater than 95% indicates that the genetic relationship between two prokaryotes is similar, and an ANI value less than 95% indicates that the strains are different [15]; therefore, the S305 strain could be identified and was classified as *E. adhaerens*. We named the strain *E. adhaerens* S305.

### Comparison of the similarity of VB_12_ synthesis pathway gene sequences between the S305 and Casida A strains and detection of VB_12_ production capacity

**Fig. 3:**
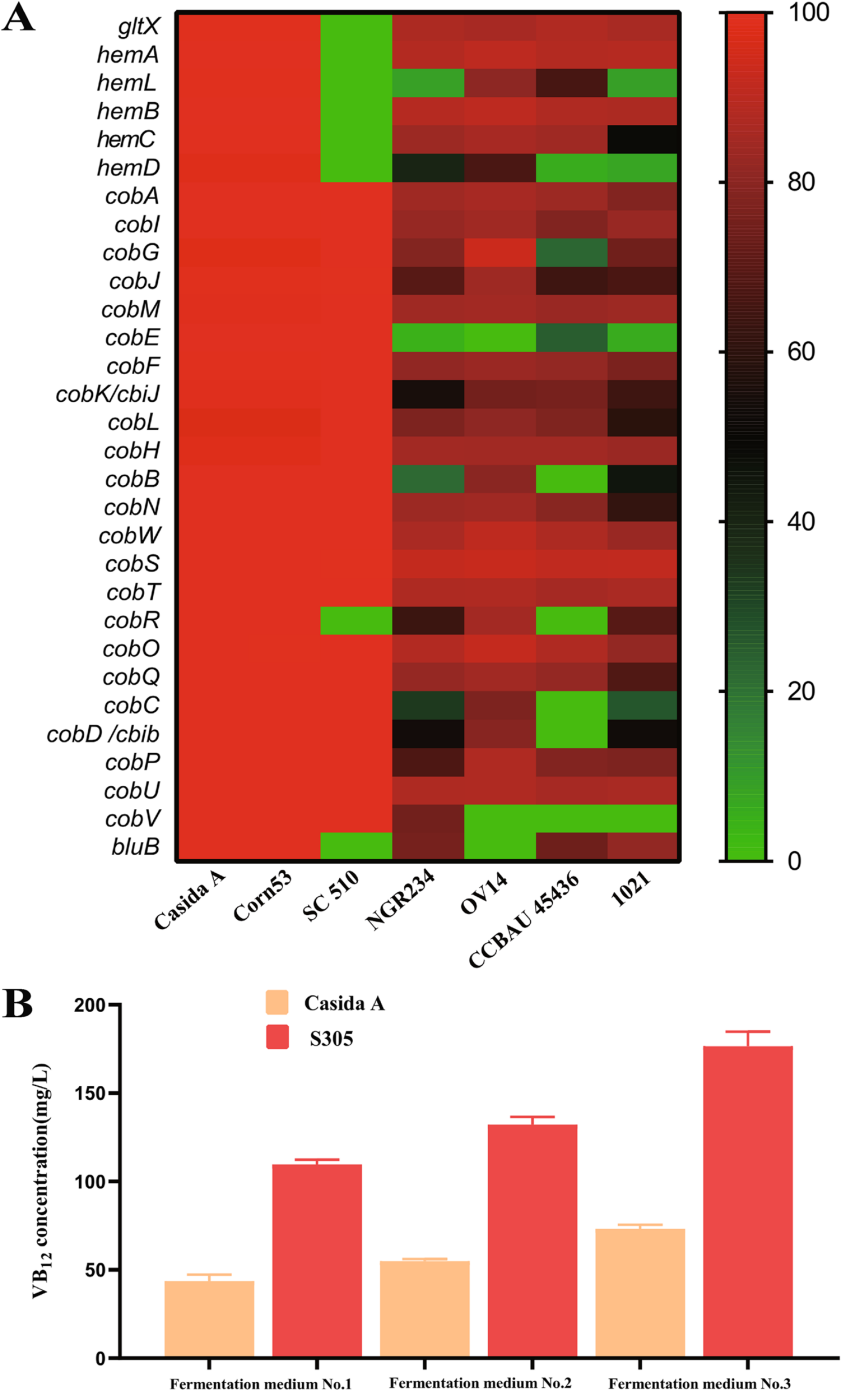
Sequence alignment analysis of the VB_12_ synthesis pathway gene and preliminary determination of the VB_12_ yield in the S305 strain. **A** Heatmap for comparison of gene sequence similarity between *Ensifer adhaerens* S305 (GenBank assembly accession GCA_029439625.1) and other strains with VB_12_ synthesis-related genes. The horizontal axis shows the strains *E. adhaerens* Casida A (GenBank assembly accession GCA_029674665.1), *E. adhaerens* Corn53 (GenBank assembly accession GCA_009883655.1), *Pseudomonas denitrificans* SC 510 (European Patent Number EP-0516647-B1), *Sinorhizobium fredii* NGR234 (NC_012587.1), *E. adhaerens* OV14 (GenBank assembly accession GCA_000583045.1), *Sinorhizobium fredii* CCBAU 45436 (GenBank assembly accession GCA_003100575.1), and *Sinorhizobium meliloti* 1021 (GenBank assembly accession GCA_000006965.1) from left to right, and the vertical axis shows the similarity of VB_12_ synthesis-related gene sequences (0–100%) from top to bottom. The "white box" indicates that the gene sequence for the strain has not been found. **B** VB_12_ production by the S305 and Casida A strains in liquid shake flask culture with different fermentation media

A total of 39 genes in the whole genome of the S305 strain are directly related to the synthesis of VB_12_, of which 30 genes are involved in the VB_12_ synthesis pathway and 9 genes are involved in the branched metabolic pathway (Table S2). The base sequence alignment results showed that the S305 strain was highly similar to Casida A, *P. denitrificans* SC 510, and *Ensifer adhaerens* Corn53 in terms of the genes related to the main VB_12_ synthesis pathway (especially the genes with a *cob* prefix), whereas the similarity of the *cob* genes between the *Ensifer* and *Sinorhizobium* strains was quite different from that between the strains of the *Ensifer* genus; VB_12_ synthesis pathway-related genes generally exist in strains of these two genera, and most of the gene sequences have high similarity (Fig. 3A). This result suggests that *Ensifer* and *Sinorhizobium* species can generally synthesize VB_12_, a finding that is consistent with the results reported by HOAN THI VU et al. [9]. Interestingly, it has been reported that the VB_12_ yield of the *P. denitrificans* SC510 strain can be improved significantly by replacing these *cob* genes [6]; these *cob* genes are highly similar to the *cob* gene sequences of the S305 and Casida A strains, and the amino acid sequences of their encoded proteins are also highly similar (Table S3). It was preliminarily speculated that the high sequence similarity of these genes with the *cob* prefix may be one of the factors contributing to the high VB_12_ yield; therefore, we purchased the Casida A strain. To compare the VB_12_ yields of the S305 and Casida A strains, the test strains were cultured in shake flasks with different medium components, and the VB_12_ yield of the S305 strain was more than double that of the Casida A strain (Fig. 3B). Therefore, we decided to further compare and analyse the two strains at the gene transcription level.

### Differences in the transcription of significantly enriched genes between the S305 and Casida A strains under the same culture conditions

**Fig. 4:**
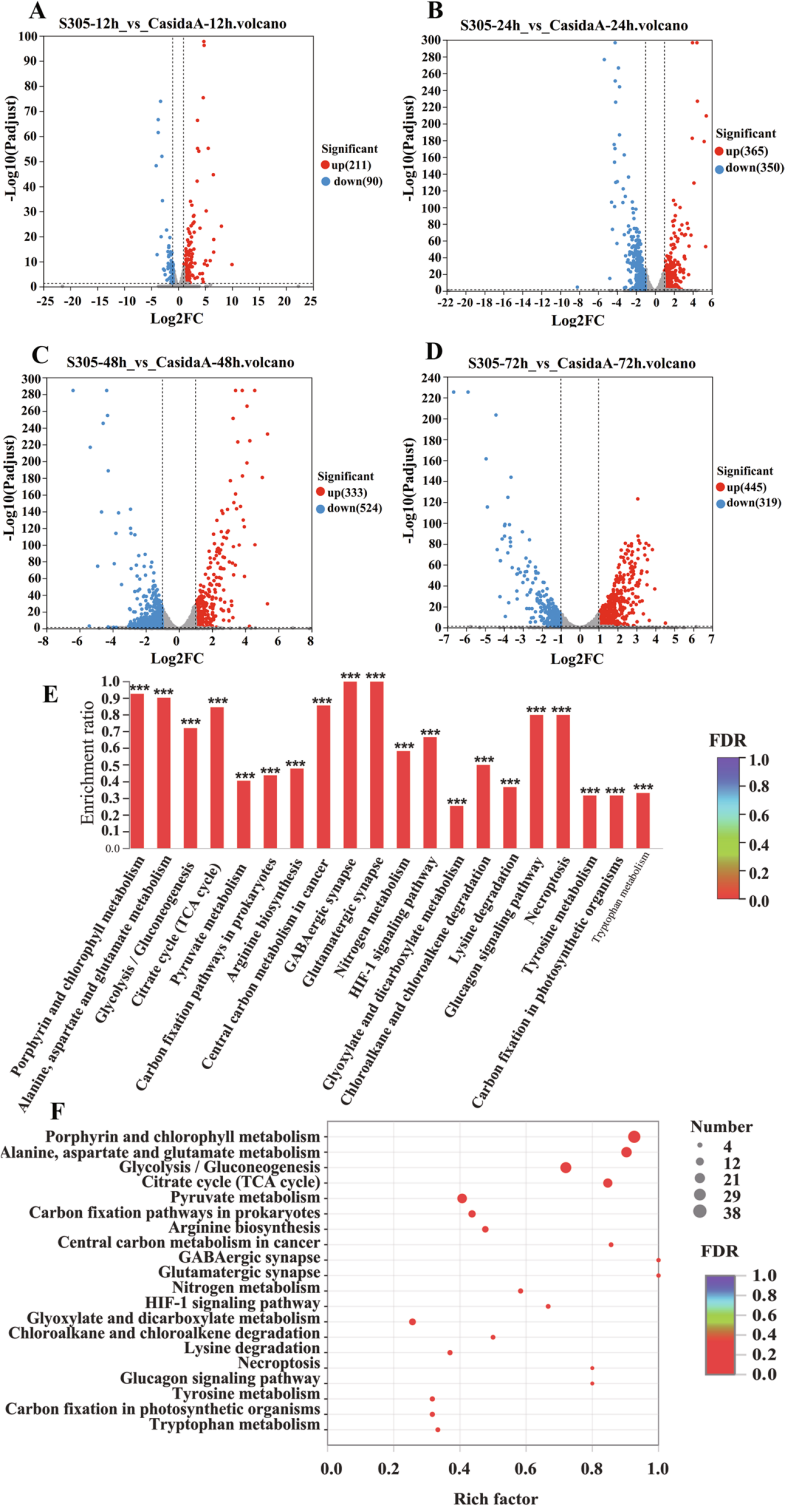
Enrichment analysis of significantly differentially expressed genes between the S305 strain and Casida A strain. **A**, **B**, **C**, **D** Volcano map of enriched genes with significant transcriptional differences between the S305 and Casida A strains during four cultivation periods (**A**-12 h, **B**-24 h, **C**-48 h, and **D**-72 h) under the same culture conditions. The blue dots represent downregulated genes, and the red dots represent upregulated genes (Padjust < 0.05, multiple change ≥ 2). **E** KEGG pathway enrichment analysis of genes with significant differences in expression in the S305 strain compared to the Casida A strain. *P* value < 0.001 is labelled as ***, *P* value < 0.01 is labelled as **, and *P* value < 0.05 is labelled as *. The colour gradient on the right side represents the *P* value. **F** The number of genes in metabolic pathways with significant differences in gene expression in the S305 strain

The results of further analyses of gene transcription in the two strains under the same culture conditions indicated that the high-quality transcriptome sequencing data of each sample exceeded 3.31 Gb, and the percentage of Q30 bases was greater than 93.8% (Table S4). The high-quality fragments (clean reads) from each sample were compared with the designated reference genomes, and the similarity ranged from 98.87% to 99.46%, indicating that the accuracy of this sequencing was high. All significantly upregulated and downregulated differentially expressed genes (DEGs) between Casida A and S305 were counted. In the S305 strain, using the Casida A strain as the comparator, 211 significantly upregulated genes and 90 significantly downregulated genes were identified after 12 h of treatment (Fig. 4A); 365 significantly upregulated genes and 350 significantly downregulated genes were identified after 24 h of treatment (Fig. 4B); 333 significantly upregulated genes and 524 significantly downregulated genes were identified after 48 h of treatment (Fig. 4C); and 445 significantly upregulated genes and 319 significantly downregulated genes were identified after 72 h of treatment (Fig. 4D). KEGG pathway correlation enrichment analysis further revealed that there were many significantly upregulated genes in the glucose metabolism and tricarboxylic acid cycle pathway, amino acid metabolism, and porphyrin metabolism pathway, all of which are closely related to VB_12_ synthesis (Fig. 4E, F).

### Analysis of transcriptionally upregulated and downregulated VB_12_ synthesis-related genes in the test strain

**Fig. 5:**
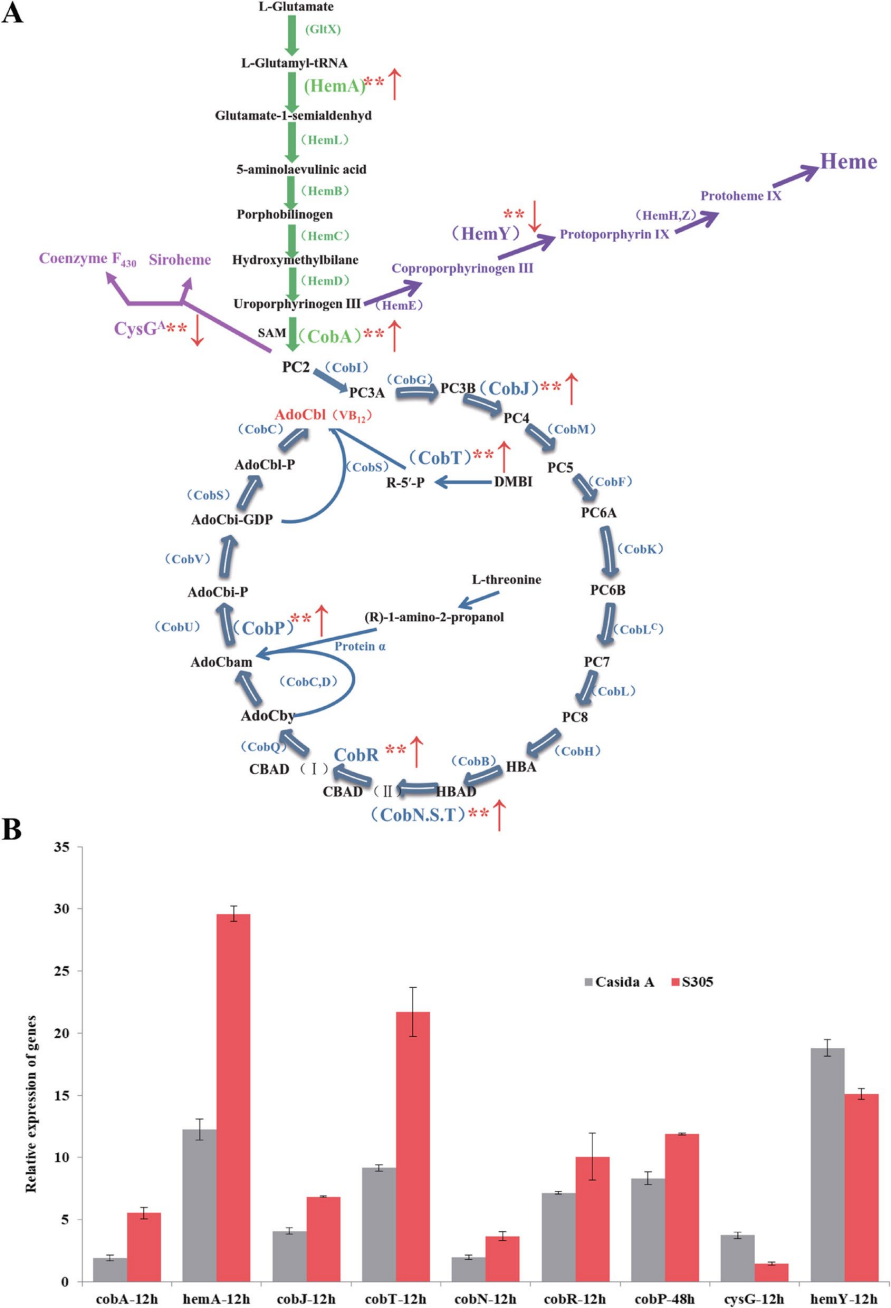
Significantly up- and downregulated genes in the VB_12_ synthesis pathway between the S305 strain and the Casida A strain. **A** Schematic diagram of the VB_12_ synthesis pathway. The asterisk in the graph represents a significant change in the transcriptional intensity of the gene in strain S305 relative to that in the Casida A strain. The up arrow represents upregulation, and the down arrow represents downregulation. *P* value < 0.01 is indicated by **. **B** Relative expression of genes significantly differentially expressed in the VB_12_ synthesis pathway between the S305 strain and the Casida A strain. The transcription level of the *cobP* gene was highest at 48 h, with the highest relative expression of other genes occurring at 12 h. The relative expression of genes was calculated using the 2-^△△T^ relative quantification method

In the S305 strain, with the Casida A strain as the comparator, 9 significantly upregulated and downregulated genes in the VB_12_ synthesis pathway were identified, including 7 significantly upregulated genes (*hemA*, *cobA*, *cobJ*, *cobT*, *cobN*, *cobP*, and *cobR*) and 2 significantly downregulated genes *(hemY* and *cysG*) (Fig. 5A); the expression levels of the genes are shown in Table S5. The expression of the 9 genes was independently verified by qPCR (Fig. 5B), and the verification results were consistent with the transcriptomic data, further demonstrating that the transcriptome sequencing data were reliable.

### Effects of the episomal overexpression of significantly upregulated genes and integrated expression on the VB_12_ yield of the strain

**Fig. 6:**
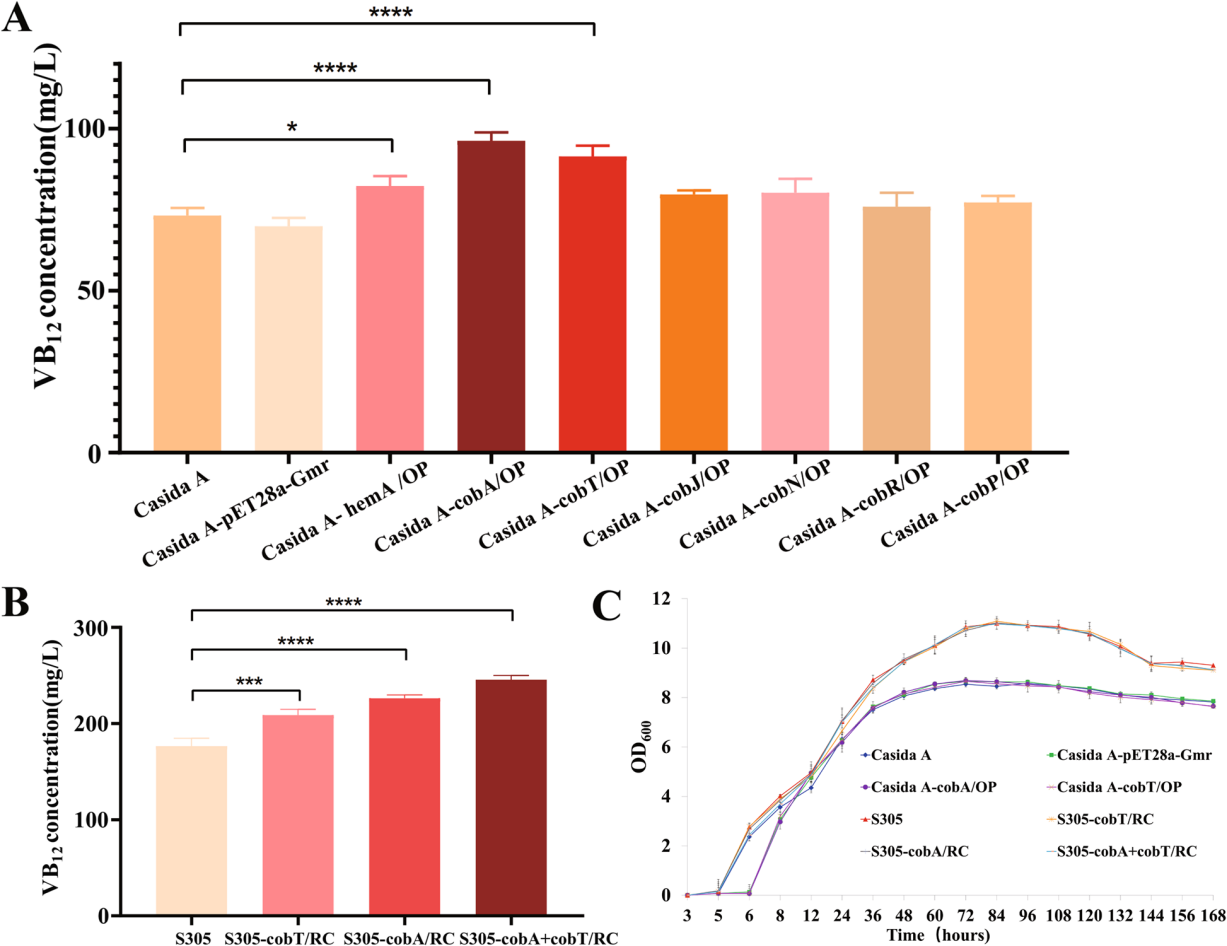
HPLC detection of VB_12_ production and growth status detection in liquid shake flask cultures for episomal expression and integrated expression strains. **A** The VB_12_ yields of the episomal overexpression strain. **B** The VB_12_ yields of the integrated expression strain. **C** Growth curves of the wild-type and recombinant strains. *P* value < 0.0001 is labelled as ****, *P* value < 0.001 is labelled as ***, *P* value < 0.01 is labelled as **, and *P* value < 0.05 is labelled as *

To verify that the abovementioned significant DEGs explained the high production of VB_12_ in the S305 strain, the Casida A strain and the Casida A-pET28a-*Gmr* strain carrying an empty overexpression plasmid were used as controls to construct seven mutant strains overexpressing the corresponding DEGs. HPLC results showed that except for the Casida A-*cobR*/OP (75.9 ± 4.31 mg/L) and Casida A-*cobP*/OP (77.2 ± 2.01 mg/L) strains, which overexpressed the *cobR* and *cobP* genes, the VB_12_ yield improved in strains that overexpressed the other genes (Fig. 6A). The Casida A-*cobA*/OP and Casida A-*cobT*/OP strains overexpressing the *cobA* and *cobT* genes had the most significant increases (yields of 96.2 ± 2.61 mg/L and 91.4 ± 3.31 mg/L, respectively, i.e., 31.4% and 24.8% higher than that of the wild-type strain (73.2 ± 2.35 mg/L)). The VB_12_ yields of the Casida A-*cobJ*/OP, Casida A-*hemA*/OP, and Casida A-*cobN*/OP strains were 79.6 ± 1.29 mg/L, 82.3 ± 3.02 mg/L, and 80.2 ± 4.29 mg/L, respectively, indirectly indicating that the higher transcription of these genes in the S305 strain compared with the Casida A strain might be related to the significantly higher VB_12_ yield. To further increase the yield of the S305 strain, *cobA* and *cobT*, the two genes with the most significant overexpression, were selected and overexpressed in the S305 strain. Importantly, episomally expressed genes are not conducive to fermentation production because of self-resistance; additionally, the loss of target traits is possible due to loss of plasmids after multiple passages [16]. Therefore, the *cobA* and *cobT* genes were integrated into the genome of the S305 strain by homologous recombination. Additionally, to ensure efficient gene expression, the strong promoter *ibpA* screened from the S305 strain was inserted upstream of the genes, and the integration sites were gene0651 and gene1218, with no chromosomal expression observed by transcriptome chromosomes. The relative expression levels of *cobA* and *cobT* in the successfully integrated strains were measured by q-PCR. The results showed that the relative expression of *cobA* and *cobT* was higher in the integrated strains than in the wild-type strains (Fig. S3), demonstrating that the integration was effective. The successfully integrated positive strains were cultured in shake flasks and assessed by HPLC. The results showed that the VB_12_ yield in the S305 strains with integration of the *cobA* or *cobT* gene alone and in the S305 strains with integration of both the *cobA* and *cobT* genes increased (Fig. 6B) in ascending order of VB_12_ yield: S305-*cobT*/RC (208.9 ± 5.92 mg/L) < S305-*cobA*/RC (226.2 ± 3.20 mg/L) < S305-*cobA* + *cobT*/RC (245.6 ± 4.36 mg/L). This yield was significantly higher than the highest reported yield of *P. denitrificans* (214.4 mg/L) [17] and the highest reported yield of *P. freudenreichii* (206.0 mg/L) [18]. Therefore, further improvement in the VB_12_ yield of the S305 strain was achieved.

### Determination of the growth status of wild-type strains and mutant strains with significantly enhanced VB_12_ yield

In addition, the growth of the above recombinant strains Casida A, Casida A-pET28a-*Gmr*, Casida A-*cobA*/OP, Casida A-*cobT*/OP, S305, S305-*cobT*/RC, S305-*cobA*/RC and S305-*cobA* + *cobT*/RC was measured (Fig. 6C), and the growth curves for the Casida A and Casida A strains with episomally expressed genes were consistent. Similarly, the growth curves for the S305 strain and the recombinant S305 strain with integrated genes were also consistent. However, the S305, S305-*cobT*/RC, S305-*cobA*/RC and S305-*cobA* + *cobT*/RC strains exhibited higher maximum growth (OD_600_) than the Casida A strain and the strains with episomally expressed genes, providing a potential explanation for why the VB_12_ yield of the S305 strain was significantly higher than that of the Casida A strain. In addition, the lag and logarithmic phases of the growth curves for the Casida A-pET28a-*Gmr*, Casida A-*cobA*/OP and Casida A-*cobT*/OP strains were significantly prolonged, a finding that may be related to the prolonged growth adaptation period of the strains due to the replacement of the gentamicin resistance gene with the recombinant plasmid. The abovementioned growth measurement results also indicate that episomal gene overexpression and integrated gene expression had little effect on the growth of the wild-type strain and demonstrate that the increase in the VB_12_ yield of strains with episomal or integrated gene expression was not caused by an increase in growth.

## Discussion

In 1980, Casida et al. discovered a bacterium that preyed on *Micrococcus luteus* and named it *E. adhaerens* Casida A in 1982 [19, 20]. The classification of *Ensifer* and *Sinorhizobium* has always been controversial. Some scholars believe that *Ensifer* should be classified as *Sinorhizobium* [21–23], and some scholars insist that all *Sinorhizobium* spp. should be renamed *Ensifer* spp. According to the *International Code of Nomenclature of Bacteria* [24], *Ensifer* is the first proposed synonym [25]. In 2008, the Judicial Commission of the International Committee on Systematics of Prokaryotes officially determined that *Sinorhizobium* and *Ensifer* are synonyms and transferred all members of the genus *Sinorhizobium* to the genus *Ensifer* [26]. However, this conclusion has not been unanimously agreed upon by rhizobial taxonomists, and most rhizobial taxonomists still do not agree with changing the name of *Sinorhizobium*. In this study, the results of a phylogenetic tree analysis showed that *Sinorhizobium* and *Ensifer* are closely related, but the comparison of ANI values indirectly indicated that the genome sequences of *Sinorhizobium* and *Ensifer* (Fig. S2) are quite different. The ANI values of the genomes of 8 *Sinorhizobium* strains and the S305 strain were between 80 and 82% (Fig. S4). The ANI values of the genomes of 8 *Ensifer* strains and the S305 strain were almost all above 95% (Fig. S5), except for a relatively low ANI value for the S305 strain and *E. adhaerens* OV14 strain (83.18%). On this basis, we speculated that the *E. adhaerens* OV14 strain is more closely related to *Sinorhizobium* strains. Hence, at the molecular level, *Sinorhizobium* and *Ensifer* can be identified as two genera.

The *hemA* gene is a key gene for the synthesis of 5-aminolevulinic acid (ALA), an important precursor of VB_12_ [27], and the *cobA* gene encodes the protein that converts uroporphyrinogen III to protocorrin-2 for the synthesis of the ring skeleton of VB_12_ [28]. Experiments have shown that the overexpression of the *hemA* and *cobA* genes can increase the yield of VB_12_ [29]. The *cobT* gene is an important gene in the salvage pathway of VB_12_ synthesis. The protein encoded by this gene can synthesize VB_12_ in two steps by catalysing the conversion of 5,6-dimethylbenzimidazole [30]; therefore, the significantly upregulated transcription of the *hemA*, *cobA*, and *cobT* genes in the S305 strain may be directly related to the high VB_12_ yield of this strain. In this study, the yield of VB_12_ improved significantly after the integrated overexpression of the *cobA* and *cobT* genes in the S305 strain. *cobJ*, *cobN*, *cobP*, and *cobR* are all genes involved in intermediate steps of VB_12_ synthesis, and the *cobJ* gene sequence in the S305 strain varies greatly from that in the Casida A strain (Table S3). The prediction of the protein encoded by this gene also showed obvious structural variation (Fig. S6). The *cobN* gene encodes a cobalt transport and chelating protein that is very important for the integration of cobalt ions in the porphyrin ring in the centre of the chemical structure of VB_12_ [31]. The upregulation of the transcription intensity of these genes may also increase VB_12_ yield. In addition, episomal overexpression of these genes increased the VB_12_ yield of the Casida A strain. *hemY* is a key gene for the synthesis of heme, which is needed for the synthesize of protoporphyrin IX from coproporphyrinogen III. Reportedly, the accumulation of coproporphyrinogen III can inhibit the activity of *hemE*, which in turn facilitates the conversion of uroporphyrinogen III to protocorrin-2, eventually leading to an increase in VB_12_ yield [32]. Transcriptomic and qPCR data showed that the transcription intensity of the *hemY* gene of the Casida A strain was significantly higher than that of the *hemY* gene of the S305 strain, potentially leading to the accelerated conversion of coproporphyrinogen III to protoporphyrin IX in the haem synthesis pathway in the Casida A strain, thereby releasing the feedback inhibition effect of coproporphyrinogen III on hemE, resulting in increased shunting of uroporphyrinogen III to haem synthesis and ultimately reducing the amount of VB_12_ synthesized. The *cysG* gene encodes siroheme synthase, which is important in another branch of VB_12_ biosynthesis. The Casida A strain exhibited significantly higher transcription of this gene than the S305 strain. Because the metabolic branch is shunted to synthesize more siroheme, VB_12_ yield in the Casida A strain may be lower, and the subsequent weakening of the *hemY* gene and the knockout of the *cysG* gene may further improve VB_12_ yield in the S305 strain. In recent years, the rapid development of synthetic biology has led to significant breakthroughs in the biosynthesis of VB_12_, which has been fully synthesized in *E. coli* [33, 34]. However, although the production of VB_12_ using *E. coli* fermentation has the advantage of a very short fermentation time, the yield is still very low (approximately 0.67–1 mg/L), and the safety of VB_12_ production using *E. coli* also needs to be further evaluated.

## Conclusion

In this study, the S305 strain was identified at the molecular level, and the genomes of *Ensifer* and *Sinorhizobium* strains were found to be significantly different through genome comparisons. This study experimentally measured the VB_12_ yield of *E. adhaerens* Casida A, which has never before been reported. Although the VB_12_ synthesis pathway genes of the *Ensifer* type strains are highly similar to the orthologues from the reported recombinant strain *P. denitrificans* SC 510, the HPLC results indirectly demonstrated that the VB_12_ synthesis pathway genes (*cob* genes) with highly similar nucleic acid and amino acid sequences were not the reason for the high VB_12_ yield of the S305 strain. Comparative transcriptomic analysis indicated that some key genes in the VB_12_ synthesis pathway may have unknown regulatory mechanisms under the same culture conditions that caused the VB_12_ yield of the S305 strain to be significantly higher than that of the Casida A strain. Episomal overexpression in the Casida A strain and integrated expression in the S305 strain also confirmed that the overexpression of these genes could improve VB_12_ yield.

These results provide guidance for screening VB_12_-producing strains for yield improvements. The biochemical metabolic pathway mechanism for VB_12_ is very clear. In recent decades, researchers in China and abroad have carried out studies on the yield optimization, purification and application of various VB_12_-producing strains, but there is still a lack of systematic studies on the high-yield expression regulation mechanisms for VB_12_. In conclusion, it is necessary to establish a relatively stable expression analysis platform and expression and knockout system for VB_12_ synthesis-related genes on the basis of comparative omics of strains with high and low VB_12_ yields to explore the key genes involved in VB_12_ synthesis, to analyse the detailed mechanism of VB_12_ synthesis and to provide an accurate and effective high-yield strategy for VB_12_-producing strains.

## Materials and methods

### Test strains and plasmids

For the strains and plasmids used in this study (Table S6).

### Test strain culture

The test strains were cultured, activated, and preserved on activated medium. The components of the activated medium were bovine heart extract powder (10 g/L), casein peptone (10 g/L), and sodium chloride (5 g/L). The medium had a pH value of 7.0. After culturing at 28 ℃ for 36 h, vigorously growing colonies were isolated and observed.

VB_12_-producing strains were cultured in liquid shake flasks (500 mL conical flasks, 200 mL medium per bottle). See Supplementary Methods 1 for the components of the fermentation medium. The strains in which each gene was episomally expressed and the strains in which each gene was integrated and expressed were cultured in No. 3 medium (Supplementary methods 1). The strains in which genes were episomally expressed were cultured with 50 µg/mL gentamicin, the strains in which the *cobA* gene was integrated and expressed were cultured with 25 µg/mL chloramphenicol, the strains in which *cobT* was integrated and expressed were cultured with 50 µg/mL apramycin, and the strains in which *cobA* and *cobT* were integrated and expressed were cultured with 25 µg/mL chloramphenicol and 50 µg/mL apramycin. The incubation temperature was 28 °C, and the flasks were shaken for 120 h prior to HPLC analysis.

### Fermentation sample processing and VB_12_ detection

The strain fermentation medium was autoclaved for 30 min. After treatment, the samples were centrifuged at 12,000 r/min for 5 min, and the supernatant was filtered through a 0.22-μm filter prior to HPLC analysis. Hydroxocobalamin was accurately weighed and dissolved in ultrapure water to prepare a 100 mg/L standard (note: VB_12_ produced by bacterial fermentation is mainly adenosylcobalamin; adenosylcobalamin is unstable and can be converted to hydroxocobalamin when exposed to light, and therefore, the standard used was hydroxocobalamin). Standards with concentrations of 10, 25, 50, and 100 mg/L were prepared for the analysis; each sample was measured three times, and the average value was used to prepare a standard curve. A WATERS 2695 high-performance liquid chromatography system coupled with a W2998 UV detector and a chromatographic column (GL InerSustain C18, 5 µM, 4.6 mm × 250 mm) was used. The settings were as follows: column temperature, 30 °C; injection volume, 10 µl; detection wavelength, 351 nm; flow rate, 1 mL/min; mobile phase A, acetonitrile; and mobile phase B, sodium acetate buffer (pH 3.6). The elution conditions were as follows: from 0 to 5 min, 10% acetonitrile gradient elution; from 5 to 10 min, 10–30% acetonitrile gradient elution.

### Whole-genome sequencing and functional annotation of the test strains

The TIANGEN whole-genome DNA extraction kit was used to extract the genomic DNA of the test strains. Purified DNA samples were sent to the ONT platform of Majorbio (Shanghai, China) for Nanopore whole-genome sequencing (third-generation whole-genome sequencing), and the whole-genome sequences of the test strains S305 and Casida A were obtained. Protein-encoding genes, repeats, and noncoding RNAs were predicted for the whole-genome DNA sequence of the test strains. The amino acid sequences of the genes were aligned with those in the GO (Gene Ontology), KEGG (Kyoto Encyclopedia of Genes and Genomes), and COG (Cluster of Orthologous Groups of Proteins) databases, and the gene and protein sequences were functionally annotated. Mauve software was used to compare the whole-genome DNA sequences of the test strains with the genome sequences of other similar strains published in the NCBI database to determine the collinearity of the genomes (Version 2.4.0, http://darlinglab.org/mauve/mauve.html) [35].

### Phylogenetic analysis of 16S rRNA and whole-genome sequences and ANI value analysis

Using the 16S rRNA sequence of the tested strain S305 as a reference, a phylogenetic tree was constructed using MEGA5 software based on the neighbour joining (NJ) method by comparison against the NCBI nucleic acid database [36]. Using the whole-genome sequence of the S305 strain as a reference, the sequencing results were compared with the NCBI database to predict similar strains, and the Fasta format genome sequences were downloaded. The whole-genome tree building software CVTree3.0 was used to construct a phylogenetic tree (Version 3.0, http://cvtree.net/v3/cvtree/index.html) [37]. Online ANI calculator software was used for whole-genome ANI analysis of strains (http://enve-omics.ce.gatech.edu/ani/index) [38]).

### Transcriptome sequencing and data analysis

The test strains were cultured in No. 3 medium in shake flasks. Ten millilitres of medium was obtained after culture for 12 h, 24 h, 48 h, and 72 h and centrifuged at 4 °C. The pellet was then transferred to a 1.5-ml sterile cryopreservation tube (repeated 3 times) and sent to Majorbio (Shanghai, China) for 2*150 bp/300 bp sequencing on an Illumina HiSeq sequencing platform. To ensure the reliability of subsequent results, the quality-controlled raw data, namely, clean data (reads), were compared with the reference genomes to obtain mapped data (reads) for subsequent analysis. Unqualified reads in the raw data were filtered out, and the clean reads obtained were used for subsequent analyses. RSEM software was used to quantitatively analyse the expression levels of the genes, and the quantitative index was FPKM (fragments per kilobase per million reads); that is, in every million sequences, each gene was measured in one thousand bases, and the number of reads was aligned. After obtaining the read counts of genes, differential expression analysis of genes between samples was performed, and p-adjust < 0.05 and |log2FC|≥ 1 were used as the thresholds for identifying DEGs between samples. Functional enrichment analysis was performed on the gene set, and KEGG pathway enrichment analysis was performed on the genes in the gene set using R script.

### Determination of relative gene expression

At similar stages of growth, a Tiangen RNA prep Pure kit was used to extract the total RNA from the two strains, and cDNA was synthesized using a reverse transcription kit (Takara Biological Company). Primer 5.0 software was used to design primers for the key enzyme-encoding genes of the VB_12_ synthesis pathway and the internal reference gene *16S rRNA* for the two strains of bacteria. The qPCR primer sequences are shown in Table S7 and were synthesized by Shanghai Sangon Biological Company. *16S rDNA* was used as the internal reference gene. The reaction volume was 20 μL, and the reaction conditions were as follows: predenaturation at 95 °C for 30 s and 40 cycles of denaturation at 95 °C for 5 s and extension at the appropriate Tm (annealing temperature of the corresponding gene) for 30 s. The cycle threshold was recorded. The relative expression level of each gene was calculated using the 2^−△△T^ relative quantitative method.

### Episomal overexpression plasmid construction and transformation

Primers were designed (Table S7), and the construction of the overexpression plasmids for all target genes was basically the same as that for the *cobT* gene, described below. The *cobT* gene was amplified using the Casida A strain genome as the template and *cobT*-28a-F and *cobT*-28a-R as the primers. The sequence fragment (783 bp) containing the gentamicin resistance gene was amplified using the pJQ200SK plasmid as the template and Gm-28a-F and Gm-28a-R as the primers. Backbone plasmid fragment 1 (719 bp) was amplified using the pET-28a plasmid as the template and P-28a-1F and P-28a-1R as the primers. Backbone plasmid fragment 2 (3798 bp) was amplified using P-28a-2F and P-28a-2R as primers. The above fragments were assembled by OE-PCR to obtain the pET28a-*Gmr*-*cobT* plasmid (Fig. S7). Other gene overexpression plasmid construction steps are described in Supplementary Methods 2. The successfully constructed overexpression plasmid was transformed into Top10 competent cells; the cells were spread onto a plate containing 50 µg/ml gentamicin and cultured overnight at 37 °C. Monoclonal strains were placed into gentamicin-containing LB liquid medium in shake flasks and cultured overnight at 37 °C. Plasmids were extracted with a Tiangen DP103 TIANprep Mini Plasmid Kit and then transformed into Casida A competent cells, which were cultured on a plate containing 50 µg/ml gentamycin at 28 °C for 36 h, after which monoclonal strains were selected. The successfully overexpressed positive clones were named Casida A-*cobA*/OP, Casida A-*cobT*/OP, Casida A-*hemA*/OP, Casida A-*cobJ*/OP, Casida A-*cobN*/OP, Casida A-*cobR*/OP, and Casida A-*cobP*/OP.

### Integrated expression plasmid construction and transformation

Primers were designed (Table S7), and the construction of all integrated expression plasmids was basically the same as that for the *cobT* gene, described below. Using the S305 strain genome as the template, the *cobT* gene fragment (1017 bp) was amplified with the primers *cobT*-200SK-F and *cobT*-200SK-R, the fragment (724 bp) containing the upstream homology arm (600 bp) was amplified with the primers UP-T200SK-F and UP-T200SK-R, the fragment (118 bp) containing the strong promoter ibpA was amplified with the primers p*ibpA*-T200SK-F and p*ibpA*-T200SK-R, and the fragment (498 bp) containing the downstream homology arm (467 bp) was amplified with the primers Down-T200SK-F and Down-T200SK-R. The gene sequence (1009 bp) containing the apramycin resistance gene (*Apr*) was amplified using the pCRISPomyces-2 plasmid as the template and the primers *Apr*-200SK-F and *Apr*-200SK-R. The backbone plasmid fragment (5551 bp) containing the sucrose lethal gene and the gentamicin resistance gene was amplified using the pJQ200SK plasmid as the template and the primers P-200SK-F and P-200SK-R. The above fragments were assembled by OE-PCR to obtain the pSK-*cobT*-*Apr* plasmid (Fig. S7). The construction steps for the *cobA* integrated expression recombinant plasmid pSK-*cobA*-*Cmr* are shown in Supplementary methods 2. The integrated expression plasmid was transformed into Top10 competent cells, which were spread onto a plate containing 50 µg/ml gentamicin and cultured at 37 °C overnight. Monoclonal strains were placed into gentamicin-containing LB liquid culture medium in shake flasks and cultured at 37 °C overnight. A Tiangen DP103 TIANprep Mini Plasmid Kit was used to extract plasmids, which were transformed into S305 competent cells. Specifically, 200 ng of plasmid was added to competent cells; the cells were shaken gently and then placed on ice for 30 min, after which they were heat shocked at 42 °C for 90 s and again placed on ice for 3 min. LB medium (600 mL) at 28 °C was added to the cells, which were incubated at 150 r/min for 1 h and then spread on *Gmr* (50 µg/ml) + *Apr* (50 µg/ml) LB solid medium and cultured at 28 °C until single colonies formed. The primary recombinant bacteria were screened for bacterial resistance to antibiotics on LB plates, and single clones were picked for subculture for a second homologous recombination. The screened primary recombination-positive single clones were transferred into *Gmr* (50 µg/ml) + *Apr* (50 µg/ml) + 15% sucrose liquid medium for three passages at 28 °C, and 1 µL of the bacterial solution was diluted 100-fold, streaked on an *Apr* (50 µg/ml) + 15% sucrose plate and cultured at 28 °C until single colonies formed. A single colony with good growth was picked for PCR using the primers *cobT*-0651-F and *cobT*-0651-R to identify the recombinant mutant strain, and the successfully identified mutant strain was named S305-*cobA*/RC. The transformation of the S305 strain with the pSK-*cobT*-Apr plasmid was performed as described in the previous section; however, the screening antibiotic apramycin (50 µg/ml) was replaced with the screening antibiotic chloramphenicol (25 µg/ml). The recombinant mutant strains were identified by PCR using the primers *cobA*-1218-F and *cobA*-1218-R, and the successfully identified mutant strain was named S305-*cobT*/RC. Finally, the pSK-*cobT*-Apr plasmid was transformed into the S305-*cobA*/RC mutant strain for screening and identification, and the successfully identified mutant strain was named S305-*cobA* + *cobT*/RC.

### Determination of the growth status of recombinant strains

The wild-type Casida A strain, the overexpression mutants Casida A-*cobA*/OP, Casida A-*cobT*/OP, Casida A-*hemA*/OP, Casida A-*cobJ*/OP, Casida A-*cobN*/OP, Casida A-*cobR*/OP, and Casida A-*cobP*/OP, and the integrated expression mutants S305-*cobA*/RC, S305-*cobT*/RC, and S305-cobA + cobT/RC were separately inoculated in 100 mL of activated culture with corresponding antibiotics at an inoculum dose of 2% and cultured at 28 °C and 150 r/min for 24 h (logarithmic growth phase); the bacteria were then transferred to 100 mL of No. 3 medium with corresponding antibiotics and cultured at 28 °C and 150 r/min for 168 h. Samples were taken every 12 h, and absorbance was measured after 3 serial tenfold dilutions (the wild-type strain was used as the control). The obtained data were analysed to draw growth curves for the strains.

### Supplementary Information

**Additional file 1: Figure S1.** Collinearity analysis of genomic nucleic acid sequence. **Figure S2.** Comparison and analysis of ANI values between the tested strain S305 and other strains. **Figure S3.** Relative expression of cobA and cobT genes in strain S305 and other recombinant strains. **Figure S4.** Comparison and Analysis of the Genome ANI between S305 and Other Sinorhizobium Strains. **Figure S5.** Comparison and Analysis of the Genome ANI between S305 and Other Ensifer Strains. **Figure S6.** Prediction of three-dimensional model for protein encoding genes related to B_12_ synthesis between S305 strain and Casida A strain. **Figure S7.** Schematic diagram of free overexpression plasmids and constructing recombinant plasmids, Take the plasmid pET28a-cobT-Gmr as an example A. The skeleton plasmid pET28a and the pET28a-cobT-Gmr overexpressed plasmid, and the kanamycin resistance of the original plasmid pET28a was replaced with gentamicin resistance. B. The plasmids of pSK-cobT-Apr and pSK –cobA-Cmr for integrated expression. **Supplementary Methods 1**. **Supplementary Methods 2**.

**Additional file 2: Table S1.** Comparison of genomic basic information between S305 and Casida A, Ensifer adhaerens Corn53 strains. **Table S2.** Ensifer adhaerens S305 genome sequencing genes and protein association annotations of VB12 synthesis related genes. **Table S3.** Similarity of amino acid sequences of the protein encoding the VB12 synthesis pathway gene between S305 and Casida A strain. **Table S4.** Quality control data statistics. **Table S5.** Significant expression of genes in the VB12 synthesis pathway of S305 and Casida A strain. **Table S6.** List of the strains and plasmids used in this study.** Table S7.** Primers used in this study.

## Data Availability

All data generated or analyzed during this study will be available from the first author (Yongheng Liu, 13519512834@139.com) for anyone who wishes to access the data.

## Abbreviations

PC n: Precorrin-n
Co-PC n: Cobalt-precorrin-n
HBA: Hydrogenobyrinic acid
HBAD: Hydrogenobyrinic acid-a,c-diamide
CBAD(II): Cob(II)yrinic acid-a,c-diamide
CBAD(I): Cob(I)yrinic acid-a,c-diamide
AdoCby: Adenosylcobyric acid
AdoCbam: Adenosylcobinamide
AdoCbi-P: Adenosylcobinamide-phosphate
AdoCbi-GDP: Adenosylcobinamide-GDP
AdoCbl-P: Adenosylcobalamin-5'-phosphate
AdoCbl: Adenosylcobalamin
DMBI: 5,6-Dimethylbenzimidazole
R-5′-P: α-Ribazole 5′-phosphate
APP: (R)-1-amino-2-propanol-O-2-phosphate
