# Supplementary material for: Research on the targeted improvement of the yield of a new VB12-producing strain, Ensifer adhaerens S305, based on genomic and transcriptomic analysis
Source: BMC Biotechnol. 2023 Dec 11;23:53. doi: 10.1186/s12896-023-00824-3 (PMC10712150; doi:10.1186/s12896-023-00824-3)
Supplement: Supplementary file 1 — Additional file 1: Figure S1. Collinearity analysis of genomic nucleic acid sequence. Figure S2. Comparison and analysis of ANI values between the tested strain S305 and other strains. Figure S3. Relative expression of cobA and cobT genes in strain S305 and other recombinant strains. Figure S4. Comparison and Analysis of the Genome ANI between S305 and Other Sinorhizobium Strains. Figure S5. Comparison and Analysis of the Genome ANI between S305 and Other Ensifer Strains. Figure S6. Prediction of three-dimensional model for protein encoding genes related to B12 synthesis between S305 strain and Casida A strain. Figure S7. Schematic diagram of free overexpression plasmids and constructing recombinant plasmids, Take the plasmid pET28a-cobT-Gmr as an example A. The skeleton plasmid pET28a and the pET28a-cobT-Gmr overexpressed plasmid, and the kanamycin resistance of the original plasmid pET28a was replaced with gentamicin resistance. B. The plasmids of pSK-cobT-Apr and pSK –cobA-Cmr for integrated expression. Supplementary Methods 1. Supplementary Methods 2. [file 12896_2023_824_MOESM1_ESM.docx]

**Supporting Information**

**Research on the targeted improvement of the yield of a new VB_12_-producing strain, *Ensifer adhaerens* S305, based on genome and transcriptome analysis**

Yongheng Liu, Wei Huang, Qi Wang, Cilang Ma, Yongyong Chang, Jianyu Su*

School of Life Science, Ningxia University, No. 539, Helan moutain-West Road, Xixia District, Yinchuan 750021, China

**
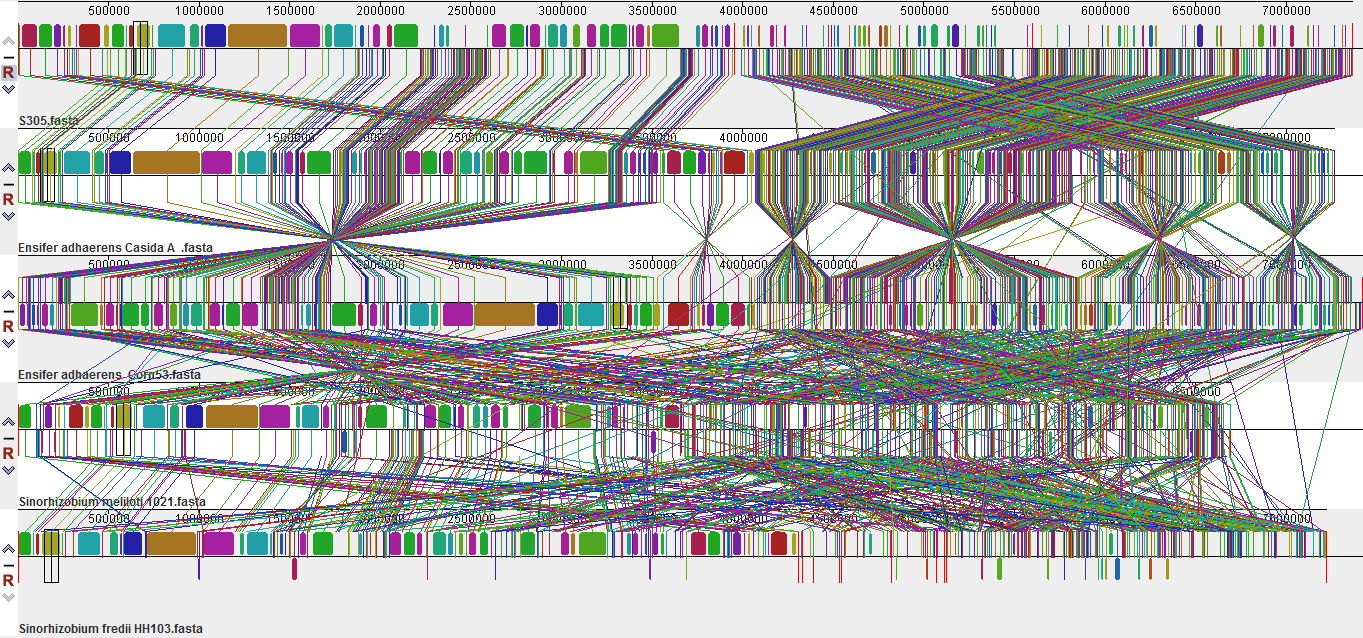
**

**Figure S1** Collinearity analysis of genomic nucleic acid sequence. The top value (0-7000000) represents the genome size (bp), and the horizontal color bar represents the distribution of gene cluster of each strain. From top to bottom, they are S305, *Ensifer adhaerens* Casida A, *Ensifer adhaerens* Corn53, *Sinorhizobium meliloti* 1021, *Sinorhizobium fredii* HH103 strain genome sequence, Vertical colored lines represent similarities between a certain genomic region and another genomic region. The higher the alignment uniformity, the more similar the two strains.


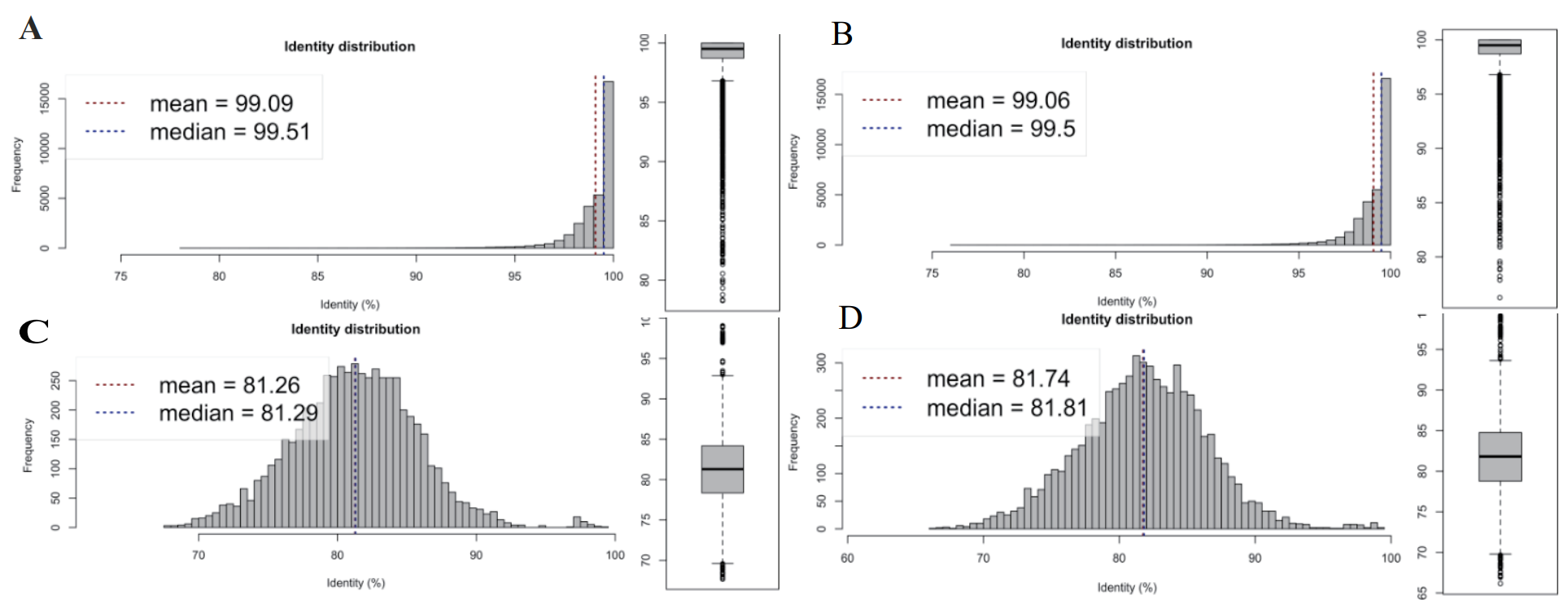


**Figure S2** Comparison and analysis of ANI values between the tested strain S305 and other strains. The 'mean' value represents the genome alignment ANI value.Figure A shows the genome comparison results between S305 and Casida A strain. Figure B shows the genome comparison results of S305 and *Ensifer adhaerens* Corn53 strain .Figure C shows the genome comparison results between S305 and *Sinorhizobium meliloti* 1021 strain .Figure D shows the genome comparison results between S305 and *Sinorhizobium fredi HH103* strain.


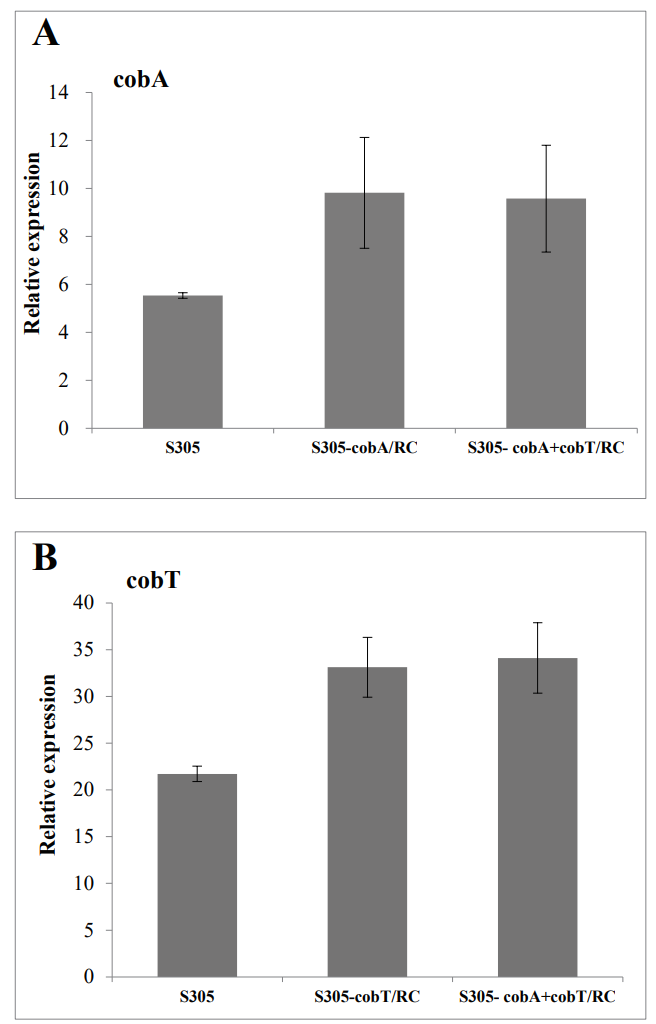


**Figure S3** Relative expression of cobA and cobT genes in strain S305 and other recombinant strains.Figure A shows the relative expression level of cobA gene in S305 and S305 cobA/RC, S305 cobA+cobT/RC strains；Figure B shows the relative expression level of cobT gene in S305 and S305 cobT/RC, S305 cobA+cobT/RC strains；


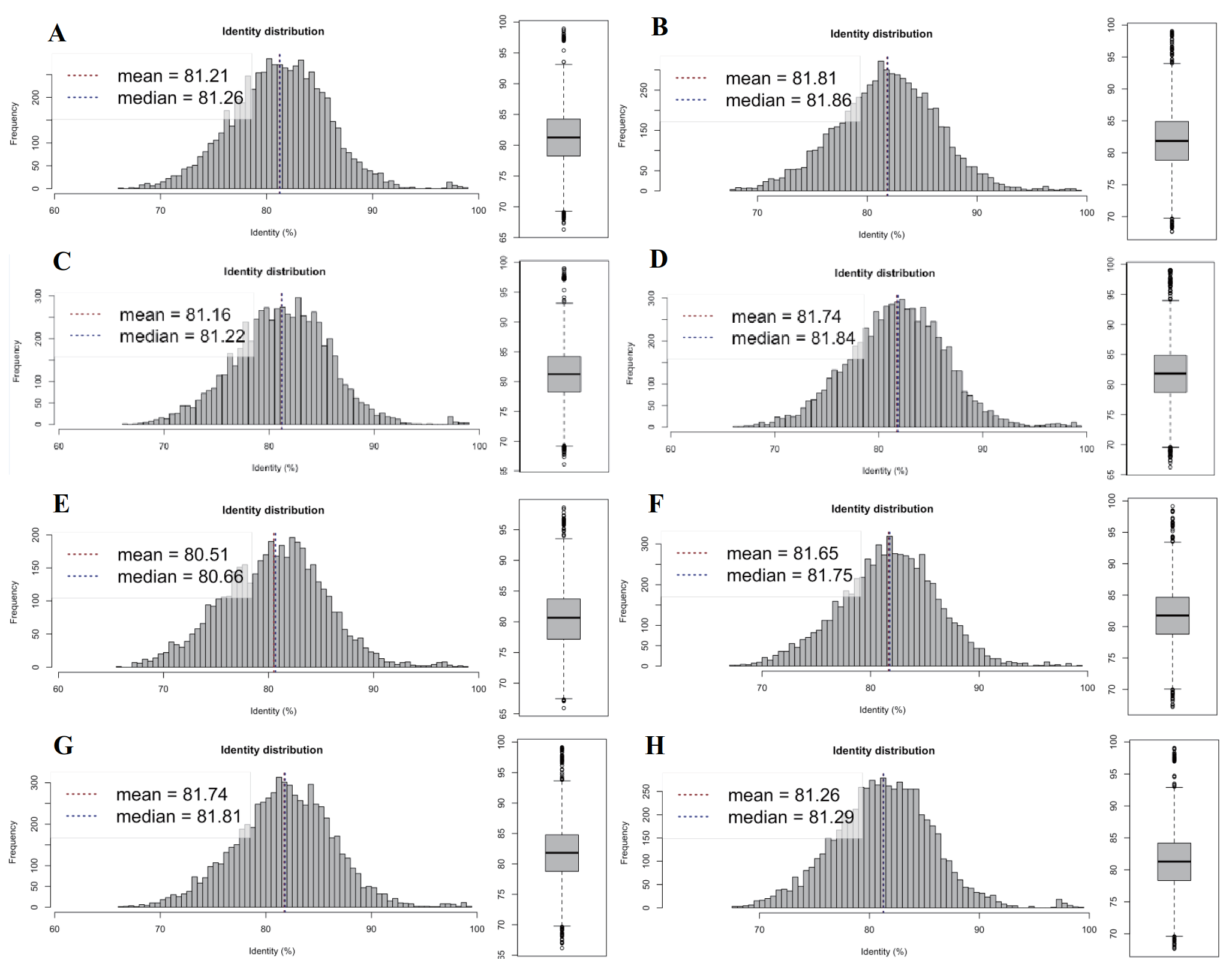


**Figure S4** Comparison and Analysis of the Genome ANI between S305 and Other Sinorhizobium Strains . The 'mean' value represents the genome alignment ANI value, (A) Genome comparison results between S305 and *Sinorhizobium meliloti* Rm41 strains. (B) Genome comparison results between S305 and *Sinorhizobium fredii* NGR234 strains. (C) Genome comparison results between S305 and *Sinorhizobium meliloti* SM11 strains. (D) Genome comparison results between S305 and *Sinorhizobium fredii* CCBAU 45436 strains. (E) Genome comparison results between S305 and *Sinorhizobium sp.* RAC02 strains. (F) Genome comparison results between S305 and *Sinorhizobium sp.* CCBAU 05631 strains. (G) Genome comparison results between S305 and *Sinorhizobium fredii* HH103 strains. (H) Genome comparison results between S305 and *Sinorhizobium meliloti* 1021 strains.


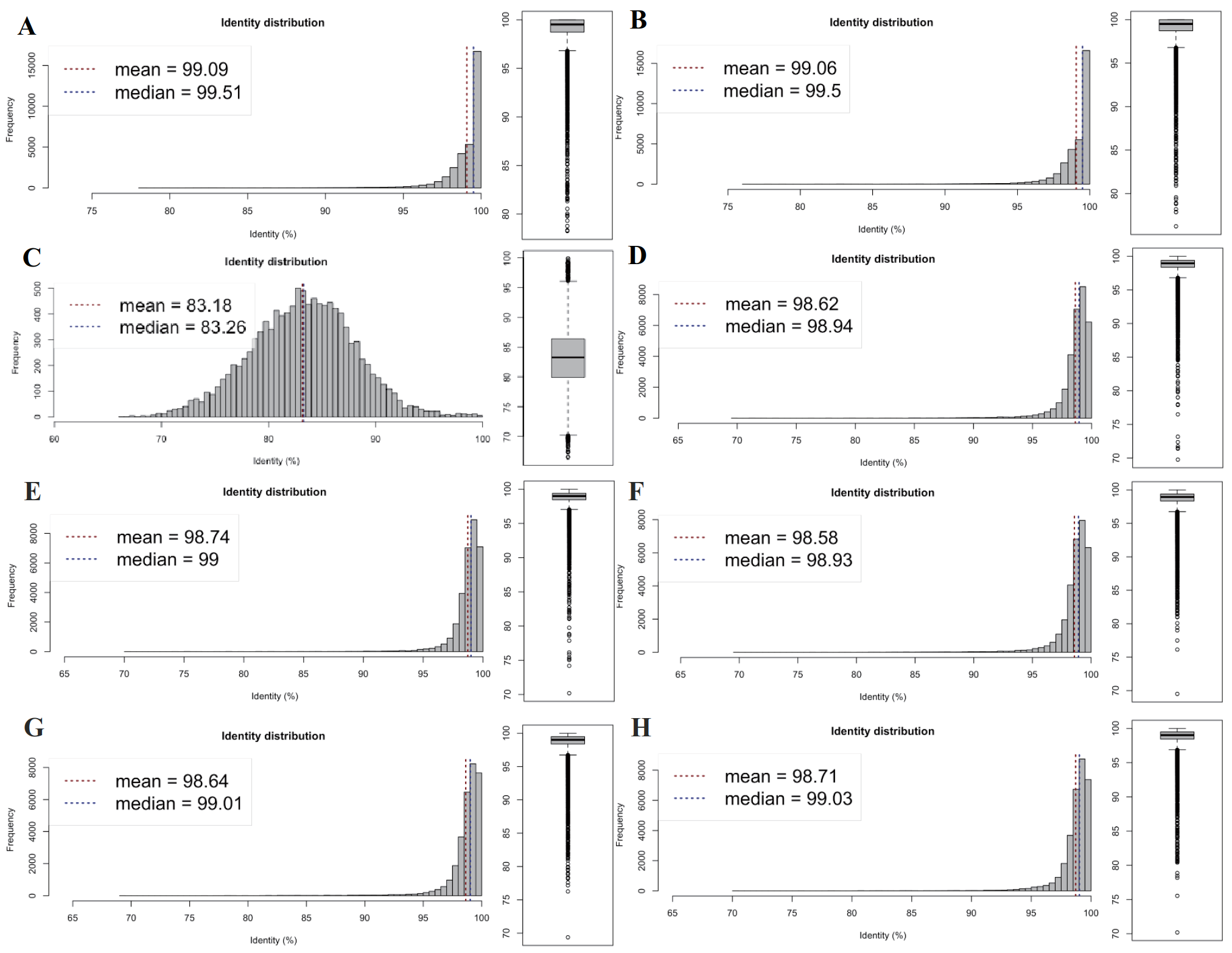


**Figure S5.** Comparison and Analysis of the Genome ANI between S305 and Other Ensifer Strains. The 'mean' value represents the genome alignment ANI value, (A) Genome comparison results between S305 and *Ensifer adhaerens* Casida A strains. (B) Genome comparison results between S305 and *Ensifer adhaerens* Corn53 strains.(C) Genome comparison results between S305 and *Ensifer adhaerens* OV14 strains. (D) Genome comparison results between S305 and *Ensifer adherens* AG1206 strains. (E) Genome comparison results between S305 and *Ensifer adhaerens* WJB133 25_10 strains. (F) Genome comparison results between S305 and *Ensifer adhaerens* X097 strains. (G) Genome comparison results between S305 and *Ensifer adhaerens* YX1 strains. (H) Genome comparison results between S305 and *Ensifer adhaerens* SD006 strains.

**Figure S6.** Prediction of three-dimensional model for protein encoding genes related to B_12_ synthesis between S305 strain and Casida A strain

### .
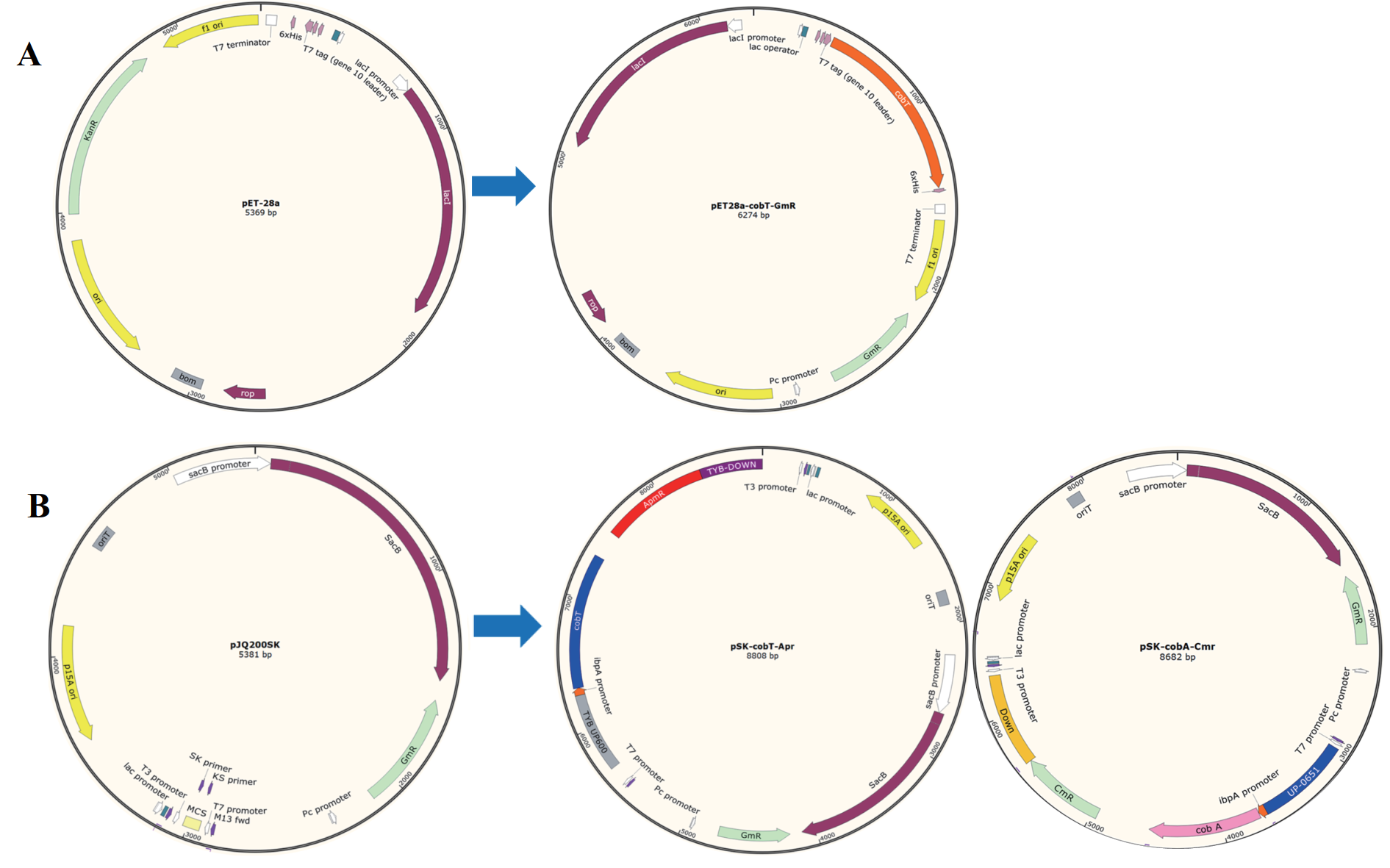


**Figure S7** Schematic diagram of free overexpression plasmids and constructing recombinant plasmids, Take the plasmid pET28a-cobT-Gmr as an example，A. The skeleton plasmid pET28a and the pET28a-cobT-Gmr overexpressed plasmid, and the kanamycin resistance of the original plasmid pET28a was replaced with gentamicin resistance.B. The plasmids of pSK-cobT-Apr and pSK –cobA-Cmr for integrated expression.

**Supplementary Methods 1**

Composition and Culture Conditions of the Fermentation Medium for Vitamin B_12_ of the Test Strain

Fermentation medium No. 1: Tryptone, 10 g L^-1^; Yeast extract, 5 g L^-1^; NaCl，10 g L^-1^; CoCl_2_•6H_2_O，0.02 g L^-1^; PH 7.0; Autoclaving, 121℃ ,30 min; Incubation Temperature ,28℃.

Fermentation medium No. 2: Tryptone, 10 g L^-1^; Yeast extract, 5 g L^-1^; NaCl, 10 g L^-1^; CoCl_2_•6H_2_O, 0.02 g L^-1^; DMBI, 0.01 g L^-1^（Add at 24 hours of cultivation）; Betaine, 5 g L^-1^; PH 7.0; Autoclaving, 121℃ ,30 min; Incubation Temperature ,28℃.

Fermentation medium No. 3: Tryptone, 17 g L^-1^; Soya peptone, 3 g L^-1^; NaCl, 5 g L^-1^; K_2_HPO_4_，2.5 g L^-1^; Glucose, 2.5 g L^-1^; CoCl_2_•6H_2_O, 0.02 g L^-1^; DMBI, 0.01 g L^-1^（Add at 24 hours of cultivation）；Betaine, 5 g L^-1^; PH 7.0; Autoclaving, 115℃ ,30 min; Incubation Temperature ,28℃.

**Supplementary Methods 2**

Construction of cobA episomal overexpression plasmid

Primers were designed (Additional file [2](https://microbialcellfactories.biomedcentral.com/articles/10.1186/s12934-023-02070-w#MOESM1): Table S7), The cobA gene was amplified using the Casida A strain genome as the template and cobA-F and cobA-R as the primers（843bp）. The sequence fragment (783 bp) containing gentamicin resistance was amplified using the pJQ200SK plasmid as the template and Gm-28a-F and Gm-28a-R as the primers. The backbone plasmid fragment 1 (719 bp) was amplified using the pET-28a plasmid as the template and P-28a-1F and P-28a-1R as the primers. The backbone plasmid fragment 2 (3798 bp) was amplified using P-28a-2F and P-28a-2R as the primers. The above fragments were assembled by OE-PCR to obtain the pET28a-Gmr-cobA plasmid (see Additional file [1](https://microbialcellfactories.biomedcentral.com/articles/10.1186/s12934-023-02070-w#MOESM1): Figure S7 for the schematic diagram of plasmid construction).

Construction of hemA episomal overexpression plasmid

Primers were designed (Additional file [2](https://microbialcellfactories.biomedcentral.com/articles/10.1186/s12934-023-02070-w#MOESM1): Table S7), The cobA gene was amplified using the Casida A strain genome as the template and hemA-F and hemA-R as the primers（1215bp）. The sequence fragment (783 bp) containing gentamicin resistance was amplified using the pJQ200SK plasmid as the template and Gm-28a-F and Gm-28a-R as the primers. The backbone plasmid fragment 1 (719 bp) was amplified using the pET-28a plasmid as the template and P-28a-1F and P-28a-1R as the primers. The backbone plasmid fragment 2 (3798 bp) was amplified using P-28a-2F and P-28a-2R as the primers. The above fragments were assembled by OE-PCR to obtain the pET28a-Gmr-hemA plasmid (see Additional file [1](https://microbialcellfactories.biomedcentral.com/articles/10.1186/s12934-023-02070-w#MOESM1): Figure S7 for the schematic diagram of plasmid construction).

Construction of cobN episomal overexpression plasmid

Primers were designed (Additional file [2](https://microbialcellfactories.biomedcentral.com/articles/10.1186/s12934-023-02070-w#MOESM1): Table S7), The cobN gene was amplified using the Casida A strain genome as the template and cobN-F and cobN -R as the primers（3831bp）. The sequence fragment (783 bp) containing gentamicin resistance was amplified using the pJQ200SK plasmid as the template and Gm-28a-F and Gm-28a-R as the primers. The backbone plasmid fragment 1 (719 bp) was amplified using the pET-28a plasmid as the template and P-28a-1F and P-28a-1R as the primers. The backbone plasmid fragment 2 (3798 bp) was amplified using P-28a-2F and P-28a-2R as the primers. The above fragments were assembled by OE-PCR to obtain the pET28a-Gmr-cobN plasmid (see Additional file [1](https://microbialcellfactories.biomedcentral.com/articles/10.1186/s12934-023-02070-w#MOESM1): Figure S7 for the schematic diagram of plasmid construction).

Construction of cobJ episomal overexpression plasmid

Primers were designed (Additional file [2](https://microbialcellfactories.biomedcentral.com/articles/10.1186/s12934-023-02070-w#MOESM1): Table S7), The cobJ gene was amplified using the Casida A strain genome as the template and cobJ-F and cobJ -R as the primers（765bp）. The sequence fragment (783 bp) containing gentamicin resistance was amplified using the pJQ200SK plasmid as the template and Gm-28a-F and Gm-28a-R as the primers. The backbone plasmid fragment 1 (719 bp) was amplified using the pET-28a plasmid as the template and P-28a-1F and P-28a-1R as the primers. The backbone plasmid fragment 2 (3798 bp) was amplified using P-28a-2F and P-28a-2R as the primers. The above fragments were assembled by OE-PCR to obtain the pET28a-Gmr-cobJ plasmid (see Additional file [1](https://microbialcellfactories.biomedcentral.com/articles/10.1186/s12934-023-02070-w#MOESM1): Figure S7 for the schematic diagram of plasmid construction).

Construction of cobR episomal overexpression plasmid

Primers were designed (Additional file [2](https://microbialcellfactories.biomedcentral.com/articles/10.1186/s12934-023-02070-w#MOESM1): Table S7), The cobR gene was amplified using the Casida A strain genome as the template and cobR-F and cobR-R as the primers（492bp）. The sequence fragment (783 bp) containing gentamicin resistance was amplified using the pJQ200SK plasmid as the template and Gm-28a-F and Gm-28a-R as the primers. The backbone plasmid fragment 1 (719 bp) was amplified using the pET-28a plasmid as the template and P-28a-1F and P-28a-1R as the primers. The backbone plasmid fragment 2 (3798 bp) was amplified using P-28a-2F and P-28a-2R as the primers. The above fragments were assembled by OE-PCR to obtain the pET28a-Gmr-cobR plasmid (see Additional file [1](https://microbialcellfactories.biomedcentral.com/articles/10.1186/s12934-023-02070-w#MOESM1): Figure S7 for the schematic diagram of plasmid construction).

Construction of cobP episomal overexpression plasmid

Primers were designed (Additional file [2](https://microbialcellfactories.biomedcentral.com/articles/10.1186/s12934-023-02070-w#MOESM1): Table S7), The cobP gene was amplified using the Casida A strain genome as the template and cobP-F and cobP-R as the primers（501bp）. The backbone plasmid fragment (5254 bp) was amplified using the pET-28a-Gmr plasmid as the template and P28a（cobP）-F and P28a（cobP）-R as the primers. The above fragments were assembled by OE-PCR to obtain the pET28a-Gmr-cobP plasmid (see Additional file [1](https://microbialcellfactories.biomedcentral.com/articles/10.1186/s12934-023-02070-w#MOESM1): Figure S7 for the schematic diagram of plasmid construction).

Construction of cobA integrated expression plasmid

Primers were designed (Additional file [2](https://microbialcellfactories.biomedcentral.com/articles/10.1186/s12934-023-02070-w#MOESM1): Table S7). Using the S305 strain genome as the template, the cobA gene fragment (847 bp) was amplified with the primers cobA-200SK-F and cobA-200SK-R, the fragment (701 bp) containing the upstream homology arm (600 bp) was amplified with the primers UP-A200SK-F and UP-A200SK-R, the fragment (115 bp) containing the strong promoter ibpA was amplified with the primers pibpA-T200SK-F and pibpA-T200SK-R, and the fragment (729 bp) containing the downstream homology arm (600 bp) was amplified with the primers Down-A200SK-F and Down-A200SK-R. The gene sequence (775 bp) containing the chloramphenicol resistance gene (Cmr) was amplified using the pHSG396 plasmid as the template and the primers Cmr-200SK-F and Cmr-200SK-R. The backbone plasmid fragment (5294 bp) containing the sucrose lethal gene and the gentamicin resistance gene was amplified using the pJQ200SK plasmid as the template and the primers P-A200SK-F and P-A200SK-R. The above fragments were assembled by OE-PCR to obtain the pSK-cobA-Apr plasmid (see the Additional file [1](https://microbialcellfactories.biomedcentral.com/articles/10.1186/s12934-023-02070-w#MOESM1): Figure S7 for the schematic diagram of plasmid construction).
